# Inhibition of Hedgehog signaling sensitizes NSCLC cells to standard therapies through modulation of EMT-regulating miRNAs

**DOI:** 10.1186/1756-8722-6-77

**Published:** 2013-10-07

**Authors:** Aamir Ahmad, Ma’in Y Maitah, Kevin R Ginnebaugh, Yiwei Li, Bin Bao, Shirish M Gadgeel, Fazlul H Sarkar

**Affiliations:** 1 Department of Pathology, Wayne State University School of Medicine, Detroit, MI 48201, USA; 2 Department of Oncology, Karmanos Cancer Institute, Wayne State University School of Medicine, Detroit, MI 48201, USA; 3 Departments of Pathology and Oncology Karmanos Cancer Institute, Wayne State University School of Medicine, 740 HWCRC Bldg, 4100 John R Street, Detroit, MI 48201, USA

**Keywords:** NSCLC, Erlotinib resistance, Hh signaling, miRNAs, EMT, GDC-0449

## Abstract

**Background:**

Epidermal growth factor receptor- tyrosine kinase inhibitors (EGFR-TKIs) benefit Non-small cell lung cancer (NSCLC) patients, and an EGFR-TKIi erlotinib, is approved for patients with recurrent NSCLC. However, resistance to erlotinib is a major clinical problem. Earlier we have demonstrated the role of Hedgehog (Hh) signaling in Epithelial-to-Mesenchymal transition (EMT) of NSCLC cells, leading to increased proliferation and invasion. Here, we investigated the role of Hh signaling in erlotinib resistance of TGF-β1-induced NSCLC cells that are reminiscent of EMT cells.

**Methods:**

Hh signaling was inhibited by specific siRNA and by GDC-0449, a small molecule antagonist of G protein coupled receptor smoothened in the Hh pathway. Not all NSCLC patients are likely to benefit from EGFR-TKIs and, therefore, cisplatin was used to further demonstrate a role of inhibition of Hh signaling in sensitization of resistant EMT cells. Specific pre- and anti-miRNA preparations were used to study the mechanistic involvement of miRNAs in drug resistance mechanism.

**Results:**

siRNA-mediated inhibition as well as pharmacological inhibition of Hh signaling abrogated resistance of NSCLC cells to erlotinib and cisplatin. It also resulted in re-sensitization of TGF-β1-induced A549 (A549M) cells as well the mesenchymal phenotypic H1299 cells to erlotinib and cisplatin treatment with concomitant up-regulation of cancer stem cell (CSC) markers (Sox2, Nanog and EpCAM) and down-regulation of miR-200 and let-7 family miRNAs. Ectopic up-regulation of miRNAs, especially miR-200b and let-7c, significantly diminished the erlotinib resistance of A549M cells. Inhibition of Hh signaling by GDC-0449 in EMT cells resulted in the attenuation of CSC markers and up-regulation of miR-200b and let-7c, leading to sensitization of EMT cells to drug treatment, thus, confirming a connection between Hh signaling, miRNAs and drug resistance.

**Conclusions:**

We demonstrate that Hh pathway, through EMT-induction, leads to reduced sensitivity to EGFR-TKIs in NSCLCs. Therefore, targeting Hh pathway may lead to the reversal of EMT phenotype and improve the therapeutic efficacy of EGFR-TKIs in NSCLC patients.

## Introduction

Lung cancer is the most common cause of cancer related mortality in the United States [1]. The primary reason for this poor outcome in non-small cell lung cancer (NSCLC) patients is the presence of systemic metastases at diagnosis in a high proportion patients [2]. Recent studies have shown that the cellular program of epithelial-to-mesencymal transition (EMT) phenotypic cells, involved in embryogenesis, is a critical step in the development of metastases. EMT is characterized by a switch from an epithelial phenotype of polarized cells with expression of epithelial markers such as E-cadherin to a mesenchymal phenotype of cells that lack polarity, are motile and have down regulation of E-cadherin. Another important characteristic of EMT cancer cells is resistance to existing cytotoxic and targeted agents, including EGFR-TKI, erlotinib. Recent data suggests that cancer cells with EMT phenotype also demonstrate stem cell like features. Pre-clinical studies suggest that measures to reverse EMT can enhance the therapeutic efficacy of erlotinib and other drugs.

The hedgehog (Hh) signaling pathway is a crucial mediator of normal organ development during embryogenesis and tissue repair during wound healing, specifically in the lung tissue. Hh pathway regulates these processes through the induction of EMT. Re-activation of the Hh pathway with induction of EMT is increasingly being implicated in carcinogenesis of many cancers. In addition, pre-clinical studies show that the inhibition of Hh pathway can reverse EMT, which in turn is associated with enhanced tumor sensitivity to cytotoxic agents. Several investigators have shown that the Hh pathway is activated in many NSCLCs. We have earlier shown that chronic exposure to TGF-β induces EMT in a NSCLC cell line A549 leading to A549 cells with greater mesenchymal features (A549M cells) [3]. Induction of EMT in these cells was associated with activation of the Hh pathway. With the knowledge that EMT is connected to drug resistance and our own observation that Hh signaling is involved in the regulation of EMT, we questioned whether inhibition of Hh signaling can reverse the drug resistance of NSCLC cells. In our current investigation, we investigated the effect of silencing of Hh signaling, using siRNA as well as pharmacological inhibitor GDC-0449, on drug sensitivity of NSCLC cells. GDC-0449 (vismodegib) is a Hh pathway inhibitor which was approved recently for the use in patients with basal cell carcinoma of the skin, a tumor type that has activating mutations in the Hh pathway. Here we report a novel role of Hh signaling in drug resistance phenotype of NSCLC cells which mechanistically involves the regulation of EMT-related microRNAs (miRNAs).

## Materials and methods

### Cell lines and reagents

The human lung adenocarcinoma cell lines A549 and H1299 were purchased from the American Type Culture Collection (Manassas, VA) and maintained according to the American Type Culture Collection’s instructions. All cells were cultured in 5% CO_2_–humidified atmosphere at 37°C. The cell lines have been tested and authenticated through the core facility (Applied Genomics Technology Center at Wayne State University) by short tandem repeat profiling using the PowerPlex 16 System from Promega. A549 cells were treated with TGF-β1 (5 ng/ml) for 21 days to generate A549M cells (EMT phenotypic cells). Cells were treated with 20 nM GDC-0449 (Genentech) for 72 hours, before conducting individual assays. Antibodies were purchased from following sources – Sox2, Nanog, EpCAM (Cell Signaling Technology, Beverly, MA) and β-actin (Sigma-Aldrich, St Louis, MO).

### Small interfering RNA (siRNA) transfection

Small interfering RNA (siRNA) specific for Hh (Shh; SHH Stealth RNAiTM siRNA) was purchased from Invitrogen. As a nonspecific control siRNA, scrambled siRNA duplex (Invitrogen) was used. Transfection was done using Lipofectamine RNAiMAX Transfection Reagent (Invitrogen) following the manufacturer’s instruction. Shh was silenced by siRNA for 48 hours prior to assay or treatment. Experiment was repeated, at least, three times independently and representative data is reported.

### miRNA transfections

Cells were seeded at 2.5 × 10^5^ cells per well in six well plates and transfected with appropriate anti-miRs/pre-miRs or miRNA-negative controls at a final concentration of 200 nM for each individual miRNA (Ambion) using DharmaFECT1 transfection reagent (Dharmacon). After 2 days of transfection, cells were split and transfected twice again before the use of these cells for specified experiments.

### Cell growth inhibition studies by MTT assay

Cells were seeded at 5 × 10^3^ cells per 100 μl of culture medium per well in 96-well plates. The number of viable cells was assessed in six wells using a 3-(4,5-dimethylthiazol-2-yl)-2,5-diphenyltetrazolium bromide (MTT) assay (Sigma). Cells were treated with Hh inhibitor (GDC-0449) for 72 hours, or with siRNA specific for Shh for 48 hours. Next, cells were treated with either Cisplatin or Erlotinib, at the specified concentrations. Control cells received equivalent amount of vehicle (0.1% DMSO) in culture medium. After treatment, cells were incubated with MTT reagent (0.5mg/ml; Sigma) at 37°C for 2 h and then with isopropanol at room temperature for 1 h. Spectrophotometric absorbance of the samples was determined by an Ultra-26 Multifunctional Microplate Reader (Tecan, Durham, NC). Cell proliferation index was calculated by assigning the vehicle-treated control cells a numerical value of 100. All the treatments, in individual experiments, therefore, represent fraction of viable proliferating cells, relative to vehicle-treated respective controls. Results were plotted as means ± SD of three separate experiments having six determinations per experiment for each experimental condition.

### Real-Time RT-PCR

For miRNA analysis, total RNA was isolated using the mirVana miRNA isolation kit (Ambion). The levels of miRNAs were determined using miRNA-specific Taqman MGB probes from the Taqman MicroRNA Assay (Applied Biosystems), as described previously [4]. The relative amounts of miRNA were normalized to internal miRNA controls RNU6B and RNU48.

### Western blot analysis

For Western blot analysis, cells were lysed in RIPA buffer containing complete mini EDTA-free protease inhibitor cocktail (Roche, Indianapolis, IN) and phosphatase inhibitor cocktails 1 and 2 (Sigma-Aldrich, St. Louis, MO) [4]. After resolution on 12% polyacrylamide gels under denaturing conditions, proteins were transferred to nitrocellulose membranes, incubated with appropriate primary / horseradish peroxidase-conjugated secondary antibodies and visualized using chemiluminescence detection system (Pierce, Rockford, IL).

### Data analysis

The experimental results presented in the figures are representative of three or more independent observations. The data are presented as the mean values ± SE. Values of *p* < 0.05 and lower were considered to be statistically significant.

## Results

### Cells with mesenchymal phenotype (A549M) are more resistant to EGFR-TKI erlotinib and cisplatin, compared to parental A549 cells

**Figure 1:**
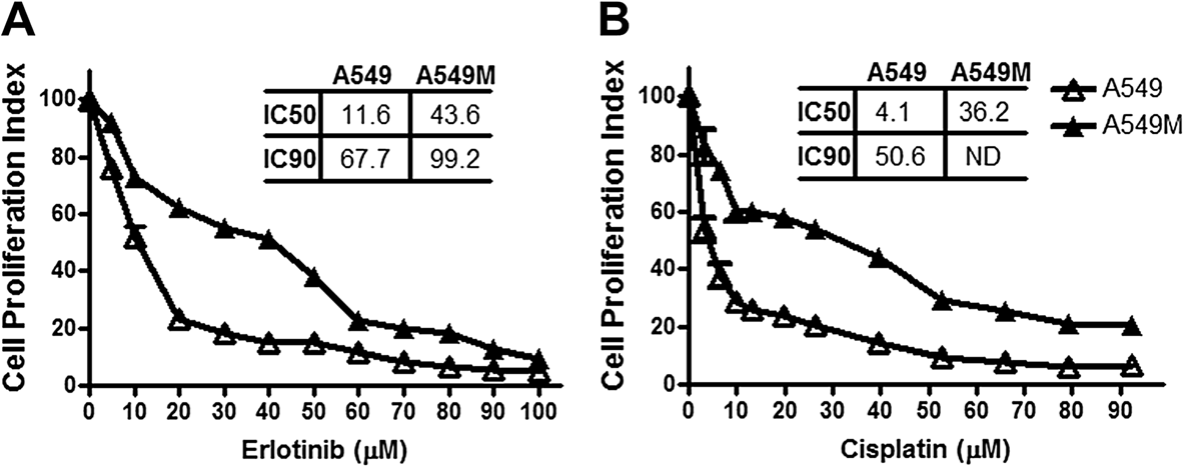
**TGF-β1-induced EMT results in drug resistance phenotype.** Dose–response curves shows that A549M cells exhibit increased cell viability, after treatment with erlotinib **(A)** and cisplatin **(B)**, compared to A549 cells. Cells were treated with indicated concentrations of erlotinib/cisplatin for 72 hours and then subjected to MTT assay. The IC50 and IC90 values for different conditions are provided in the table within the individual figures. *ND*: IC90 could not be determined. *p<0.05.

EMT phenotypic cancer cells have been shown to acquire drug resistance [5-8]. Our earlier data established that A549 cells with mesenchymal phenotype (A549M cells) acquire invasiveness *in vitro* as well as *in vivo*[3], and, therefore, we started our current investigation with the hypothesis that A549M cells should be more resistant to therapeutic drugs because of their mesenchymal phenotype. To test this hypothesis, we treated A549 and A549M cells with increasing doses of erlotinib and cisplatin for 72 h, and measured cell viability. We found significantly higher number of proliferating A549M cells than A549 cells (p<0.05) at all the tested doses of erlotinib (Figure 1A) as well as cisplatin (Figure 1B), suggesting that A549M cells are indeed more resistant to erlotinib or cisplatin, consistent with the EMT phenotype. The IC50 values as well as the IC90 values for A549M cells were significantly higher for erlotinib (Figure 1A) and cisplatin (Figure 1B), further confirming their drug resistance characteristics.

### Inhibition of hedgehog signaling sensitizes mesenchymal A549M cells to erlotinib and cisplatin

**Figure 2:**
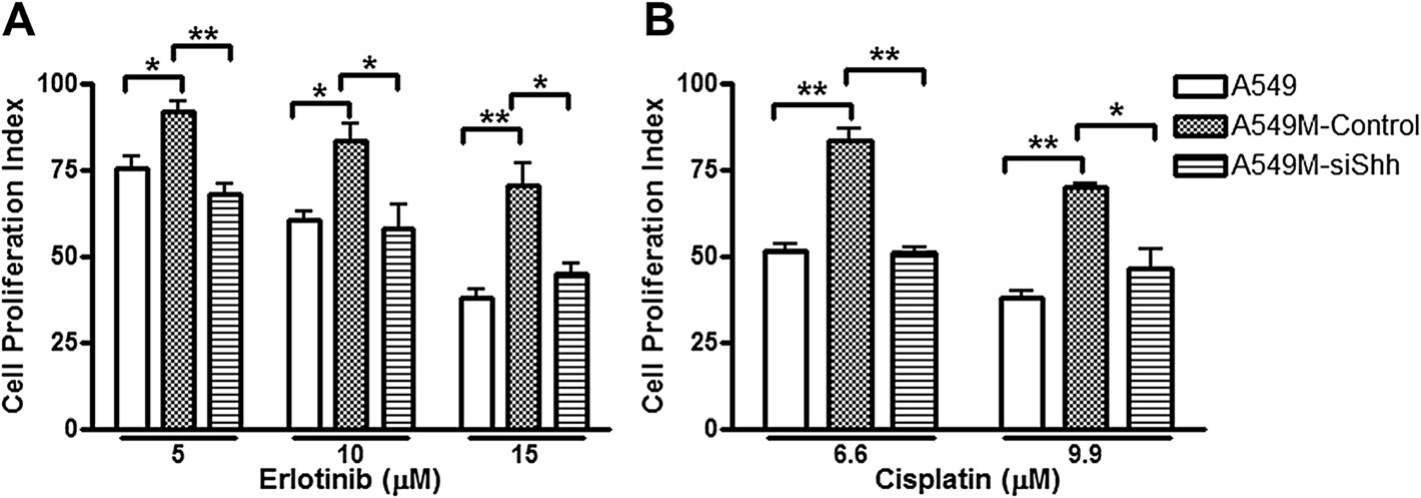
**Knock-down of Shh sensitizes A549M cells to standard therapies.** Cell proliferation of A549M cells was significantly reduced after treatment with erlotinib **(A)** and cisplatin **(B)** following Shh knock-down. Cells were first treated with vehicle (A549M-control) or with specific si-RNA against Shh (A549M-siShh) for 48 hours and then with indicated concentrations of erlotinib/cisplatin for 24 hours. Parental A549 cells were included as a control to verify the induced resistance of A549M cells to erlotinib/cisplatin. All the plotted values are relative to vehicle-treated A549 cells. *p<0.05 and **p<0.01.

**Figure 3:**
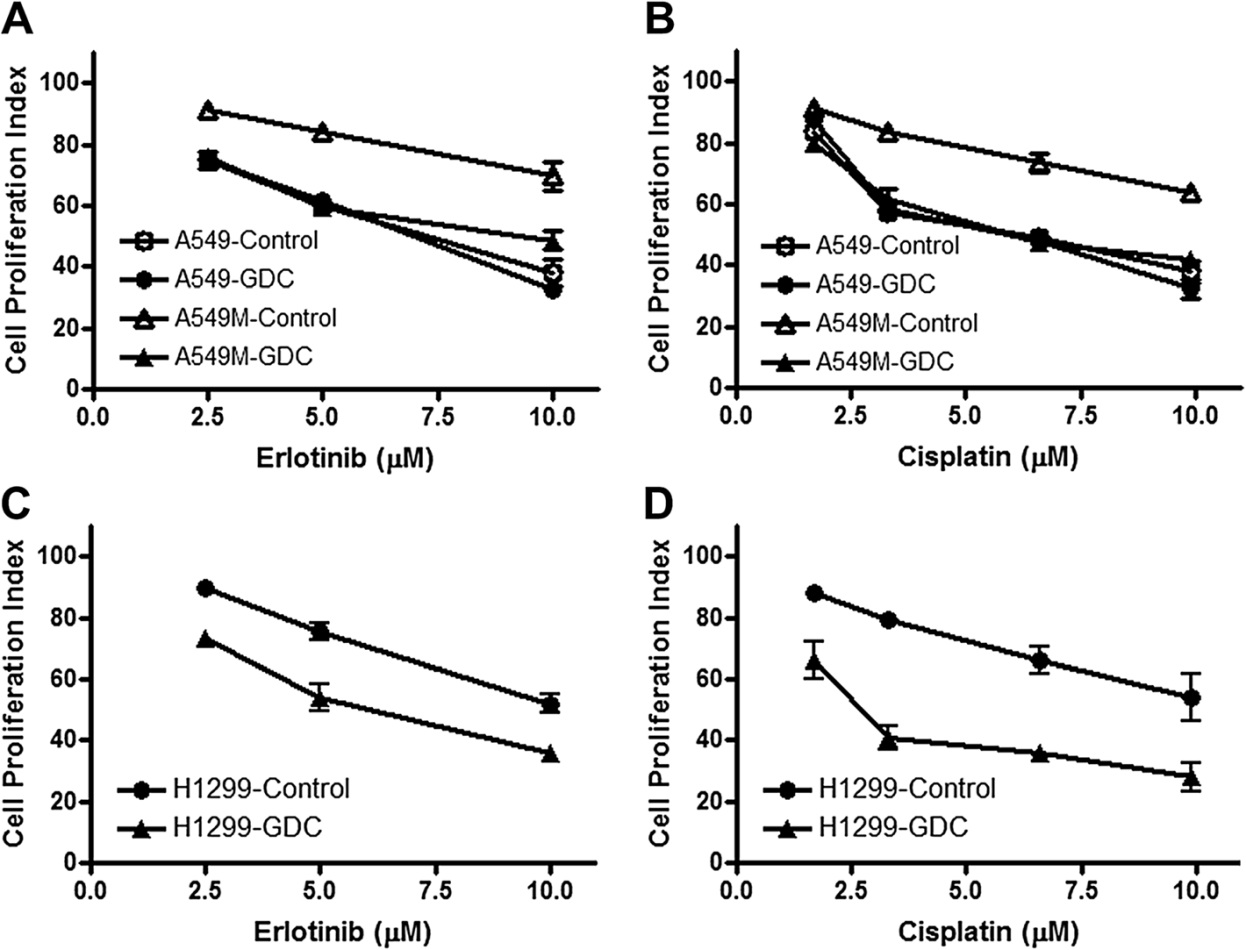
**Hedgehog inhibitor, GDC-0449 (GDC) sensitizes A549M as well as H1299 cells to standard therapies.** Pre-treatment with GDC-0449 (20nM) markedly reduced cell proliferation of A549M cells (A549M-GDC) **(A-B)** as well as H1299 cells (H1299-GDC) **(C-D)**, compared to vehicle treated respective control cells, when they were exposed to erlotinib or cisplatin for 72 hours. Control A549 cells did not exhibit such sensitization **(A-B)**. All the plotted values are relative to vehicle-treated cells.

Next, we evaluated whether Hedgehog (Hh) inhibition can sensitize A549M cells to erlotinib or cisplatin. We first used siRNA approach and inhibited Shh, a ligand of the Hh pathway to test whether the knock-down of Shh sensitizes A549M cells to erlotinib and cisplatin. A549M cells were transfected with Shh-specific siRNA, control cells were transfected with scrambled siRNA and the cells were treated with erlotinib or cisplatin. Additionally, parental A549 cells were included in the experiment to confirm comparatively increased resistance of A549M cells to erlotinib and cisplatin. As previously shown [3], siRNA against Shh was found to significantly down-regulate the expression of Shh. A549M cells with Shh knock-down showed significant reduction in cell proliferation (p<0.05) when treated with erlotinib (Figure 2A) and cisplatin (Figure 2B). To confirm the impact of inhibition of Hh signaling on drug resistance, we treated A549M cells with pharmacological inhibitor GDC-0449 for 72 h, followed by treatment with erlotinib or cisplatin, and the cell viability was assessed after 72 h of treatment. A549M cells were more resistant to erlotinib and cisplatin, compared to parental A549 cells, and A549M cells treated with GDC-0449 showed reduced cell proliferation (Table 1), as evidenced by lower IC_50_ of both the drugs in the cells pre-treated with GDC-0449. This suggests that Hh inhibitor GDC-0449 sensitizes mesenchymal phenotypic cells to standard therapy. The results of GDC-0449 sensitization of A549M cells, at a few selected doses of erlotinib (Figure 3A) and cisplatin (Figure 3B) clearly show that the A549M cells pre-treated with GDC-0449 are more sensitive to the drugs. It is interesting to note that GDC-0449 was not able to sensitize the parental A549 cells (Figure 3A-B),which may be due to the fact that the parental cells do not express appreciable levels of Shh.

**Table 1:** GDC-0449 lowers the IC-50 of erlotinib/cisplatin in A549M / H1299 cells

**Cell Line**	**Standard Therapy**	**IC50 (μM)**		**% Decrease in IC50**
		**Without GDC**	**With GDC**	
A549	Erlotinib	11.56	11.27	2.51
	Cisplatin	4.11	4.04	1.70
A549M	Erlotinib	43.64	15.76	63.89
	Cisplatin	36.16	9.64	73.34
H1299	Erlotinib	10.57	7.20	31.90
	Cisplatin	12.15	4.19	65.56

Cells were pre-treated with 20nM GDC-0449 (GDC) for 72 h or vehicle control, prior to treatments with increasing doses of erlotinib or cisplatin for 72 h.

For further validation of this finding, we performed experiments using another cell line, the H1299 cell line, a NSCLC cell line with mesenchymal phenotype. H1299 cells are known to be resistant to erlotinib and cisplatin [9-11]. These cells were treated with GDC-0449 for 72 h prior to treatments with erlotinib or cisplatin for 72 h, similar to the experiments with A549M cells above. H1299 cells pretreated with GDC-0449 showed reduced cell proliferation when exposed to erlotinib (Figure 3C) or cisplatin (Figure 3D) as well as reduced IC_50_ (Table 1), compared to untreated H1299 cells, and these results are consistent with the results obtained from A549M cells (Figure 2, Table 1). We also confirmed the specific role of the Hh ligand Shh in drug sensitivity of H1299 cells by using Shh siRNA knock-down prior to treatment with cisplatin or erlotinib. H1299 cells with Shh knock-down showed decreased cell viability (Results not shown), confirming the involvement of Shh in restoring sensitivity to standard therapy. Furthermore, we treated H1299 cells with GDC-0449 alone and combined the data with the observations in Figure 3 to analyze the combined effects of GDC-0449 and erlotinib/cisplatin. As shown in Table 2, the observed values significantly exceeded the expected theoretical values indicating a sensitizing effect of GDC-0449, as opposed to a mere additive effect. Together, these results indicate that treatment of NSCLCs with Hh inhibitor prior to or concurrent with standard therapy could improve sensitivity of NSCLCs with EMT features.

**Table 2:** GDC-0449 sensitizes H1299 cells to erlotinib/cisplatin

**Erlotinib (A)10 μM**	**GDC (B)20 nM**	**Erlotinib + GDC [Expected (A+B)]**	**Erlotinib + GDC [Observed]**
48.00 ± 1.8	12.81 ± 0.7	60.81 ± 1.9	68.60 ± 1.1
**Cisplatin (C)9.9 μM**	**GDC (B)20 nM**	**Cisplatin + GDC [Expected (C+B)]**	**Cisplatin + GDC [Observed]**
46.14 ± 3.1	12.81 ± 0.7	58.95 ± 2.8	71.93 ± 2.4

The inhibition by erlotinib **(A)** and cisplatin **(C)** was calculated from the experiment shown in Figure 3C-D and all the values represent % Inhibition of H1299 cell proliferation under specified treatments. Erlotinib/cisplatin as well as GDC-0449 (GDC) **(B)** inhibited cell proliferation individually and the combination was significantly more effective.

### TGF-β1-induced EMT of NSCLC cells involves modulation of Cancer Stem Cells (CSCs) and miRNAs

**Figure 4:**
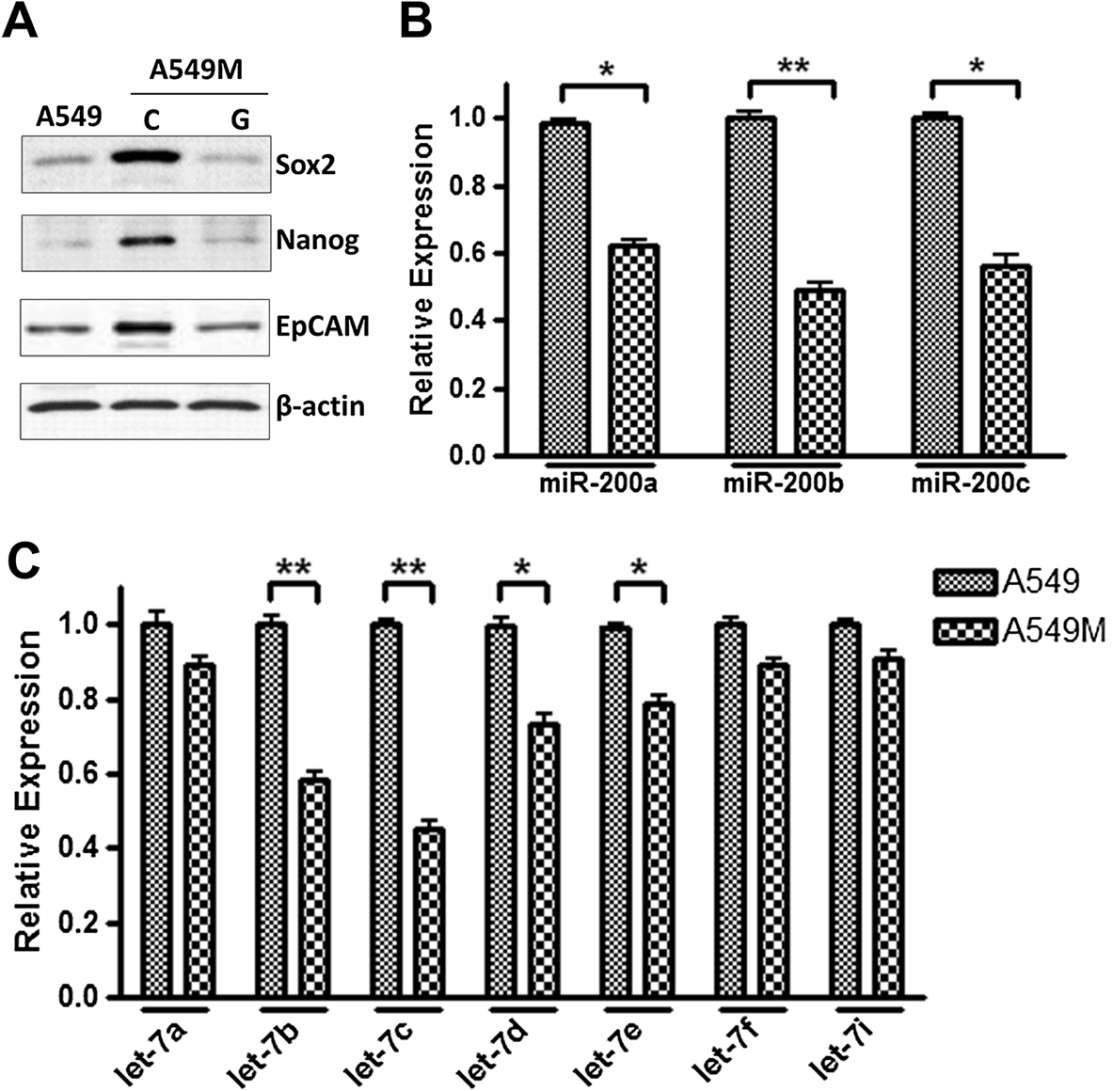
**Modulation of CSC markers and miRNAs accompanies EMT of NSCLC cells. (A)** A549M cells exhibit increased expression of CSC markers Sox2, Nanog and EpCAM and GDC-0449 inhibited such TGF-β-induced expression of CSC markers. TGF-β1-induced EMT also involved changes in the expression levels of **(B)** miR-200 family and **(C)** let-7 family of miRNAs. RNU6B and RNU48 were used as miRNA controls against which the data was normalized. *p<0.05 and **p<0.01.

In order to fully understand the mechanism(s) of drug resistance that accompany induction of EMT in NSCLC cells, we investigated CSC markers (Sox2, Nanog and EpCAM) and the expression levels of several EMT-related miRNAs in parental A549 vs. mesenchymal A549M cells. A comparison of levels of CSC markers, by western blot analysis, revealed that the protein levels of CSC markers are elevated in vehicle-treated control A549M cells (Figure 4A). Since our earlier work has established a mechanistic role of Hh signaling in EMT of these cells, we treated A549M cells with GDC-0449 to inhibit Hh signaling and found that, compared to levels in vector-treated A549M cells, GDC-0449-treated cells had significantly reduced levels of CSCs (Figure 4A). As further molecular signature of mesenchymal A549M cells, we investigated some miRNAs that have been implicated in the EMT of cancer cells. We chose two families of miRNAs, the miR-200 and let-7 families, and our data revealed that several member miRNAs of these EMT-regulating miRNA families are down-regulated in the mesenchymal cells (Figure 4B-C). In particular miR-200b and let-7c miRNAs were found to be the most significantly down-regulated miRNAs from the two respective families. These results are consistent with the documented epithelial phenotype promoting role of these two miRNA families.

### Re-expression of selected miRNAs can reverse TGF-β1-induced drug resistance

**Figure 5:**
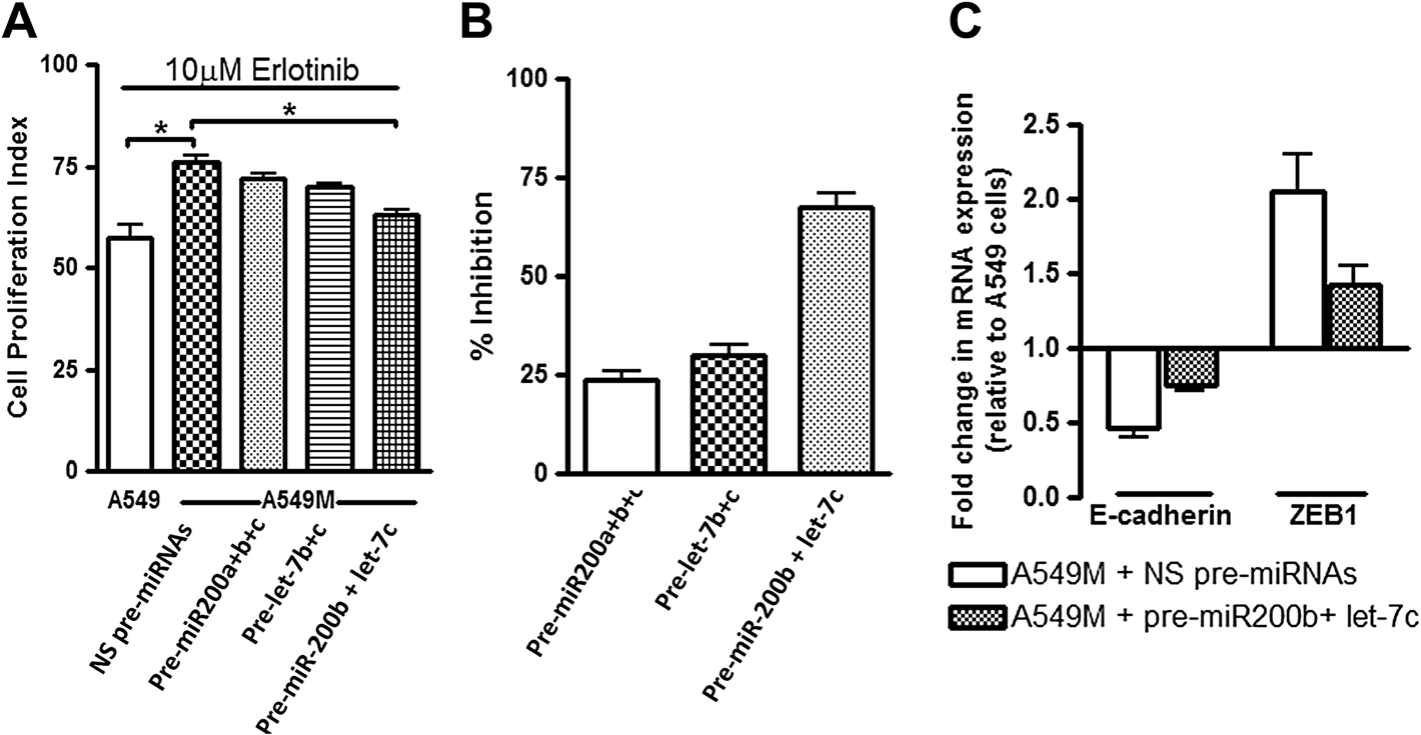
**Mechanistic role of miRNAs in TGF-β1 induced drug resistance. (A)** Re-expression of miR-200s and let-7s sensitized A549M cells to erlotinib treatment. **(B)** Data from Figure 5A was used to calculate the extent of sensitization by re-expression of miRNAs upon erlotinib treatment, as measured by % inhibition of A549M resistance compared to parental A549 cells. **(C)** Re-expression of miR-200b+let-7c reversed EMT. E-cadherin and ZEB1 mRNA levels were determined by real time RT-PCR using GAPDH as the internal control. All the plotted values in Figure 5A are relative to vehicle-treated A549 cells. RNU6B and RNU48 were used as miRNA controls against which the data was normalized. *p<0.05.

Having observed differential expression of several miRNAs in parental A549 vs. A549M cells, we next assessed whether these miRNAs are mechanistically involved in the drug resistance associated with the TGF-β1-induced mesenchymal phenotype. Since the response to erlotinib and cisplatin was similar in our earlier experiments, we chose erlotinib for these mechanistic studies. A549M cells were transfected with pre-miRNAs for the re-expression of chosen miRNAs and to test whether re-constitution of these miRNAs can reverse the drug resistance. We found that the re-expression of different miRNAs did reverse the drug resistance of A549M cells (Figure 5). Firstly, we transfected A549M cells with a cocktail of pre-miR-200a+pre-miR-200b+pre-miR-200c and observed 23.77% inhibition of TGF-β1-mediated effect on erlotinib resistance (Figure 5A-B). From the let-7 family, we chose let-7b and let-7c for re-expression because they were the most-down-regulated miRNAs from their family in A549M cells. Re-expression of these miRNAs resulted in slightly more inhibition (29.76%) of TGF-β1-mediated effect on erlotinib resistance (Figure 5A-B). Finally, we re-expressed the top most down-regulated miRNAs from both families and transfected A549M cells with a cocktail of pre-miR-200b+pre-let-7c. We found much more potent inhibition (67.69%) of TGF-β1-mediated effect on erlotinib resistance (Figure 5A-B). We also confirmed the reversal of EMT by pre-miR-200b+let-7c treatment and the results of real time RT-PCR are shown as Figure 5C. Pre-treatment with miR-200b+let-7c significantly abrogated the inhibition of E-cadherin expression and also reduced ZEB1 levels (Figure 5C), all of which are indications of the reversal of EMT.

### miRNAs that reverse TGF-β1-induced drug resistance also play a role in GDC-0449’s inhibition of erlotinib resistance

**Figure 6:**
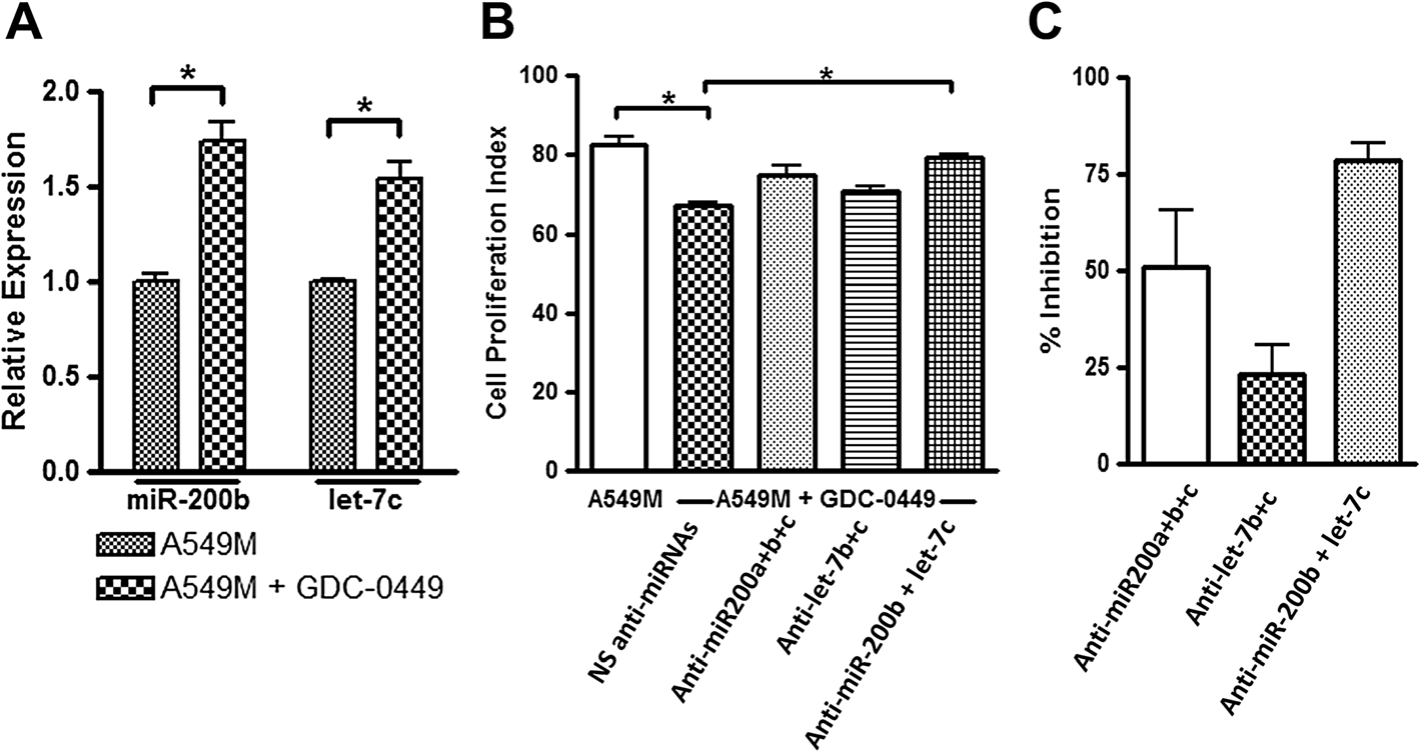
**Mechanistic role of miRNAs in GDC-0449 effects on drug resistance. (A)** GDC-0449 up-regulated the expression of miR-200b and let-7c, the two top regulated miRNAs in their respective families. **(B)** Down-regulation of miR-200 and let-7 family miRNAs abrogated the GDC-0449 mediated inhibition of erlotinib resistance in A549M cells. **(C)** Data from Figure 6B was used to calculate the abrogation of GDC-0449 effect through the use of anti-miRNAs, as measured by % inhibition of A549M sensitivity to GDC-0449, compared to control A549M cells. All the plotted values in Figure 6B are relative to vehicle-treated A549M cells and the first bar in the same figure represents A549M cells treated only with 10μM erlotinib. RNU6B and RNU48 were used as miRNA controls against which the data was normalized. *p<0.05.

Our results thus far indicated a role of miR-200b and let-7c in TGF-β1-induced EMT that results in resistance to erlotinib. With our focus on mechanistic involvement of Hh signaling in this process, we next tested the effect, if any, of GDC-0449 on these miRNAs. Exposure to GDC-0449 for 72 h resulted in a significant up-regulation (p<0.05) of both the miRNAs in A549M cells (Figure 6A) which might explain the increased sensitivity of cells to erlotinib after GDC-0449 treatment. To verify this, we down-regulated miRNAs, by using commercially available specific anti-miRs, in GDC-0449 treated A549-M cells, followed by treatment with erlotinib. We found that the down-regulation of miRNAs abrogated the GDC-0449-induced sensitization of A549M cells to erlotinib treatment (Figure 6B). Whereas down-regulation of miR-200 family abrogated GDC-0449 effect by 51.06%, let7-b/c could abrogate this effect by only 23.40% (Figure 6C). Down-regulation of miR-200b+let-7c was found to be the most effective with 78.72% inhibition of GDC-0449 effect (Figure 6C).

## Discussion

The major findings of our study are – a) TGF-β1-induced EMT of NSCLC cells leads to increased resistance to both erlotinib and cisplatin; b) Hh signaling seems to play a role in such EMT-induced drug resistance because siRNA-mediated as well as pharmacological down-regulation of Hh signaling inhibits resistance to both the drugs and c) EMT regulating miRNAs such as miR-200b and let-7c are mechanistically involved in Hh signaling- and EMT-mediated resistance of NSCLC cells to TKI erlotinib.

Resistance to currently available anti-cancer drugs such as erlotinib represents a major clinical challenge [12]. A number of investigational tools such as gene expression, xenograft assays and proteomic profiling techniques have been used to find biomarkers associated with sensitivity to erlotinib in a panel of sensitive and insensitive NSCLC cell lines [9,13,14]. These studies reported that sensitive cell lines express well-established epithelial markers E-cadherin and catenin as well as exhibit the typical cobblestone epithelial morphology and tight cell–cell junctions of epithelial cells. On the other hand, insensitive cell lines express characteristics of mesenchymal cells, including the expression of vimentin, fibronectin, and ZEB1, consistent with more fibroblastic scattered morphology. In summary, these results are suggestive of the process of EMT as an indicator of sensitivity to EGFR inhibitors. Moreover, a study by Prudkin *et al.* has indicated an association with these EMT markers in the sequential pathogenesis of squamous cell carcinoma [15], suggesting that the combination of EGFR-TKI with the inhibitor of EMT-inducing-molecules could become a novel approach toward the treatment of lung cancer, especially for NSCLC. The hedgehog (Hh) signaling pathway is involved in embryogenesis particularly in the development of the lungs. This pathway is not active in adult tissues but it can be activated in many cancers including NSCLC [16-19]. In addition, blocking Hh signaling inhibits the growth, invasion and metastasis of cancer cells, which is associated with the down-regulation of Snail and up-regulation of E-cadherin. Also, over-expression of GLI1, the effector molecule of the Hh signaling pathway, in epithelial cells, leads to an aggressive phenotype with down-regulation of E-cadherin [20,21]. All of this evidence suggests a connection between Hh signaling and EMT which can potentially be exploited for therapy.

Based on the available literature discussed above, there seems to be a correlation between EMT, drug resistance and Hh signaling but the mechanistic details of this inter-relationship is not clearly understood. We have previously shown that there is a transcriptional up-regulation of Shh by TGF-β1 as a key step during the induction of EMT in NSCLC cell line [3]. As the next step, we now provide evidence in support of the role for Hh signaling pathway in drug resistance phenotype of NSCLC cells that accompanies the processes of EMT. Our results show an increase in resistance to drugs when EMT is induced in NSCLC cells that are chronically exposed to TGF-β1. Resistance was enhanced to both cisplatin and erlotinib. A similar response of EMT cells to these two different drugs suggests a broader role of EMT in drug resistance that might not be confined to any particular class of anti-cancer drugs.

With the increased resistance of EMT cells to drugs, reversal of EMT for the re-sensitization of such cells is very intuitive. The challenge, however, lies in the elucidation of the regulation of EMT which can potentially help identify novel targets for therapy and reversal of EMT. Taking a cue from our previous work, we investigated Hh signaling in relation to EMT-induced drug resistance. As a proof-of-principle, we inhibited Shh by siRNA in NSCLC cells that had undergone EMT, and this resulted in re-sensitization of NSCLC cells to erlotinib and cisplatin. To make our results clinically relevant, we used a pharmacological inhibitor of Hh signaling, GDC-0449, and obtained very similar results. These results clearly demonstrate the relevance of inhibition of Hh signaling for reversal of EMT and overcoming drug resistance. In addition to the TGF-β1-induced EMT as a model, we confirmed our results in H1299 cells that have a dominant mesenchymal phenotype and also exhibit elevated levels of Shh. Re-sensitization of H1299 cells to erlotinib and cisplatin was observed after treatment with GDC-0449 further supports our hypothesis that reversal of EMT through down-regulation of Hh signaling is an effective strategy to overcome drug resistant phenotype.

Since acquired resistance to conventional therapies is a major clinical concern, re-sensitization of tumors offers a viable alternative in the absence of novel therapeutic options. Different ‘sensitizing’ agents have been investigated for their ability to reverse drug resistance [22-25]. Of interest, re-sensitization to erlotinib [26-28] as well as cisplatin [24,29] has been demonstrated. In a recent study [24], miR-98 has been shown to sensitize cisplatin-resistant human lung adenocarcinoma cells. The miR-98 belongs to let-7 family of miRNAs and was down-regulated in resistant cells. These results are in agreement with our own observations where we found reduced levels of let-7 family members in erlotinib and cisplatin resistant cells. In a very recent report [30], the role of let-7c in determining docetaxel resistance in lung cancer model has been described. This further provides evidence in support of the role of miRNAs, particularly let-7c in a broader drug resistance phenotype with functional implications, and these results are consistent with our findings using a different class of drugs. In addition to let-7 family, we observed down-regulation of miR-200 family and, collectively, this underlines a role of EMT-regulating miRNAs in erlotinib/cisplatin resistance. In experiments involving combination of agents/drugs, a distinction between additive vs. sensitization effects is always a concern. The combined effects of Hh inhibition and erlotinib/cisplatin were found to be significantly more than the individual or simple additive effects, which is reminiscent of sensitization. Furthermore, pre-treatment of resistant A549M cells with GDC-0449 significantly lowered the IC_50_ values of erlotinib and cisplatin, almost to the levels of sensitive parental A549 cells, which further confirms re-sensitization.

We observed increased expression of CSC markers and modulation of miRNAs (miR-200s and let7s) in NSCLC cells with TGF-β1-induced EMT. The role of CSCs in drug resistance of lung cancer cells has been demonstrated [31,32]. Our results showed a significant down-regulation of CSC markers Sox2, Nanog and EpCAM upon inhibition of Hh signaling in A549-M cells by GDC-0449, which provided direct evidence in support of the connection between Hh signaling and CSCs in a model system with induced EMT. Further, miR-200 and let-7 families of miRNAs are well known inhibitors of EMT [4,33,34] and the data on growth, invasion and metastasis of lung cancer cells [10,35-37] fully supports their established biological activity. As expected, we observed down-regulation of these miRNAs in TGF-β1-treated cells (A549M cells). Re-expression of these miRNAs, especially re-expression of the most down-regulated miRNAs, miR-200b and let-7c, inhibited the TGF-β1-mediated resistance of NSCLC cells to erlotinib. Interestingly, we observed a direct induction of these two-miRNAs by Hh inhibitor GDC-0449 treatment. Additionally, re-expression of these two miRNAs significantly reversed EMT markers. This could explain the observed inhibition of TGF-β1-induced effects by GDC-0449. It appears that TGF-β1 mediated induction of EMT is in part mediated by down-regulation of miR-200 and let-7 family miRNAs and contributes to drug resistance. The ability of GDC-0449 to maintain the levels, through direct up-regulation of these miRNAs, abrogates the TGF-β1-induced EMT, resulting in drug resistance. It is also interesting to note that the modulation of multiple members of the same miRNA family, either miR-200 family or the let-7 family, did influence the TGF-β1/GDC-0449 effects but not to the same extent as the combination of miR-200b and let-7c. This can probably be explained by the fact that multiple members of the same miRNA family have overlapping target genes and concurrently targeting miRNAs from different families can be more effective through their combined effects on wide range of mutually exclusive targets.

In summary, our present studies established a mechanistic role of Hh signaling in EMT-associated drug resistance phenotype of NSCLC cells which is mediated through novel regulation of CSCs and the EMT. Therefore, the inhibition of Hh signaling could be a useful approach for reducing tumor aggressiveness in NSCLC, and as such, the reversal of EMT, particularly through modulation of miRNAs, could also be useful for re-sensitization of drug-resistant NSCLC cells to conventional therapeutics, which would likely contribute to improved survival of patients who rightfully deserve better treatment outcomes.

## Abbreviations

CSC: Cancer stem cells; EGFR: Epidermal growth factor receptor; EMT: Epithelial-to-mesenchymal transition; Hh: Hedgehog; NSCLC: Non-small cell lung cancer; Shh: Sonic hedgehog; TKI: Tyrosine kinase inhibitor; miRNA: microRNA.

## Competing interest

SMG has served on advisory board and speaker bureau for Genentech. For the remaining authors, none was declared.

## Authors’ contribution

AA designed and performed experiments, analyzed data and drafted manuscript; MYM performed experiments and analyzed data; KRG, YL and BB performed part of the experiments; SMG designed study and edited manuscript; FHS designed and supervised study, provided lab resources and reagents, and edited manuscript. All authors read and approved the final manuscript.
